# Optimization of Tumor Treating Fields (TTFields) Frequency and Treatment Duration in Colorectal Cancer Cells

**DOI:** 10.1002/cam4.70976

**Published:** 2025-05-19

**Authors:** Menglan Liu, Zhaoran Su, Paul‐Philipp Hagemann, Malte Fischer, Michael Linnebacher

**Affiliations:** ^1^ Molecular Oncology and Immunotherapy, Clinic of General Surgery University Medical Center Rostock Rostock Germany; ^2^ Department of Gastrointestinal Surgery People's Hospital of Tongling City Tongling China

**Keywords:** cell viability, colorectal cancer, frequency optimization, tumor treating fields

## Abstract

**Introduction:**

Colorectal cancer (CRC) is one of the most prevalent malignancies worldwide. Tumor Treating Fields (TTFields), a novel anticancer therapy using intermediate frequency and low‐intensity alternating electric fields, has demonstrated efficacy in various cancers, but its application in CRC remains underexplored.

**Objective:**

To determine the optimal frequency and treatment duration of TTFields therapy for CRC through in vitro experiments.

**Methods:**

Four human CRC cell lines (2 MSI: HROC110 T1 M7, HROC285 T0 M2; 2 MSS: HROC450, HROC463) were treated using the inovitro TTFields system. Frequencies (100, 200, 300 kHz) and daily exposure durations (16, 20, 24 h/day) were varied. Cell viability was assessed after 72 h using crystal violet staining and spectrophotometry. Data were analyzed using *t*‐tests or ANOVA, with *p* < 0.05 considered statistically significant.

**Results:**

TTFields significantly reduced cell viability at all tested frequencies. The 100 kHz frequency yielded the most pronounced effects, especially in MSI cell lines (*p* < 0.001). Treatment duration of 24 h/day led to the greatest viability reduction across all cell lines (*p* < 0.01), while 16 h/day and 20 h/day showed comparatively weaker effects.

**Conclusion:**

Our study demonstrates that TTFields effectively reduce CRC cell viability, supporting their therapeutic potential. Further studies are needed to understand their mechanisms and synergy with existing treatments

## 1 Introduction

Colorectal cancer (CRC) is one of the most prevalent malignancies worldwide, with its high incidence and mortality rates representing a significant public health challenge [1]. Despite advancements in treatment modalities like surgical resection, radiotherapy, and chemotherapy, managing advanced or recurrent CRC remains especially challenging. The recent introduction of Tumor Treating Fields (TTFields) represents a novel approach to cancer therapy [2, 3, 4]. TTFields involve the application of intermediate frequency and low intensity alternating electric fields which can interfere with the mitosis of tumor cells, thereby inhibiting tumor growth.

The efficacy and safety of TTFields have been demonstrated across various cancer types, including glioblastoma, mesothelioma, and non‐small cell lung cancer, with promising results [5, 6]. Voloshin et al. [7] reported that applying TTFields to human ovarian cancer cell lines in vitro significantly led to a significant reduction in cell counts compared to untreated cells, with effectiveness depending on both frequency and intensity. However, the application of TTFields in CRC remains in the exploratory phase, particularly lacking baseline studies on in vitro experimental parameters. Optimizing these parameters in laboratory settings will help identify the most effective treatment conditions and lay the ground for further investigations into TTFields mechanisms.

The primary objective of this study was to determine the optimal frequency and treatment duration of TTFields therapy for CRC through in vitro experiments and advance the clinical application of this innovative therapy.

## 2 Materials and Methods

### 2.1 Cell Lines and Culture

In this study, four human CRC cell lines from our biobank were utilized, including HROC110 T1 M7, HROC285 T0 M2, HROC450, and HROC463. These cell lines were derived from CRC patients and exhibited either microsatellite‐instability (MSI) or microsatellite‐stability (MSS). Specifically, HROC110 T1 M7 and HROC285 T0 M2 are MSI, while HROC450 and HROC463 are MSS. Most cell lines were established using pre‐established patient‐derived xenograft (PDX) models, following the methodology detailed in our previous report [8]. They were cultured in DMEM/Ham's F‐12 medium (Catalog No. DMEM‐12‐A, Capricorn Scientific, Ebsdorfergrund, Germany) supplemented with 10% fetal bovine serum (Catalog No. P40‐37500, PAN‐Biotech, Aidenbach, Germany) and were maintained in a humidified incubator at 37°C with 5% CO_2_ in accordance with standard laboratory protocols. The culture medium was replaced every 2–3 days, and the cells were passaged when they reached approximately 80% confluence. All cell lines utilized in this study have undergone short tandem repeat profiling to prove authenticity and were mycoplasma‐negative.

### 2.2 TTFields Treatment

TTFields treatment was administered using the inovitro system (Novocure, Haifa, Israel), which utilizes low‐intensity alternating electric fields in the 50 to 500 kHz frequency range. The fields were applied to ceramic dishes containing embedded insulated arrays, allowing uniform field distribution. The base plates are connected to TTFields generators, which are controlled by a computer with inovitro software. On the first day of the experiment, cells in the logarithmic growth phase were seeded at a density of 100,000 cells/ml in 2 mL per well onto coverslips in standard 6‐well plates. The plates were incubated for 24 h to allow cell attachment and growth. The following day, the coverslips containing the attached cells were carefully transferred to ceramic dishes on base plates, and the dishes were covered with parafilm, then placed in a CO_2_ incubator set to 20°C for TTFields treatment. Frequencies (100 kHz, 200 kHz, 300 kHz) and daily usage times (24‐h/day, 20‐h/day, 16‐h/day) were varied to assess their effects. The experimental groups were exposed to TTFields for 3 days, while the control groups were incubated at 37°C without electric field exposure. Each experiment was repeated three times.

### 2.3 Cell Viability Assessment

Following the completion of the treatment regimen, the viability of the cells was evaluated through the utilization of the crystal violet staining method. The cells were fixed with 4% paraformaldehyde for 15 min, followed by staining with a 0.2% crystal violet solution (catalog number 548‐62‐9, Merck, Darmstadt, Germany) for 2 h. The excess stain was removed by washing with water, and the stained cells were solubilized in 1% sodium dodecyl sulfate (Catalog No. 151‐21‐3, Merck). The absorbance was then measured at 570 nm using a spectrophotometer. The cell viability was calculated using the following formula: Cell viability (%) = (Absorbance of living treated cells−Absorbance of blank) / (Absorbance of living control cells−Absorbance of blank) × 100.

### 2.4 Statistical Methods

All experimental data are presented as mean ± standard deviation (SD). The statistical significance of differences between groups was assessed using either *t*‐tests or analysis of variance (ANOVA). A *p*‐value of less than 0.05 was considered to indicate a statistically significant result.

## 3 Results

### 3.1 Impact of Different Frequencies on Cell Viability

**FIGURE 1:**
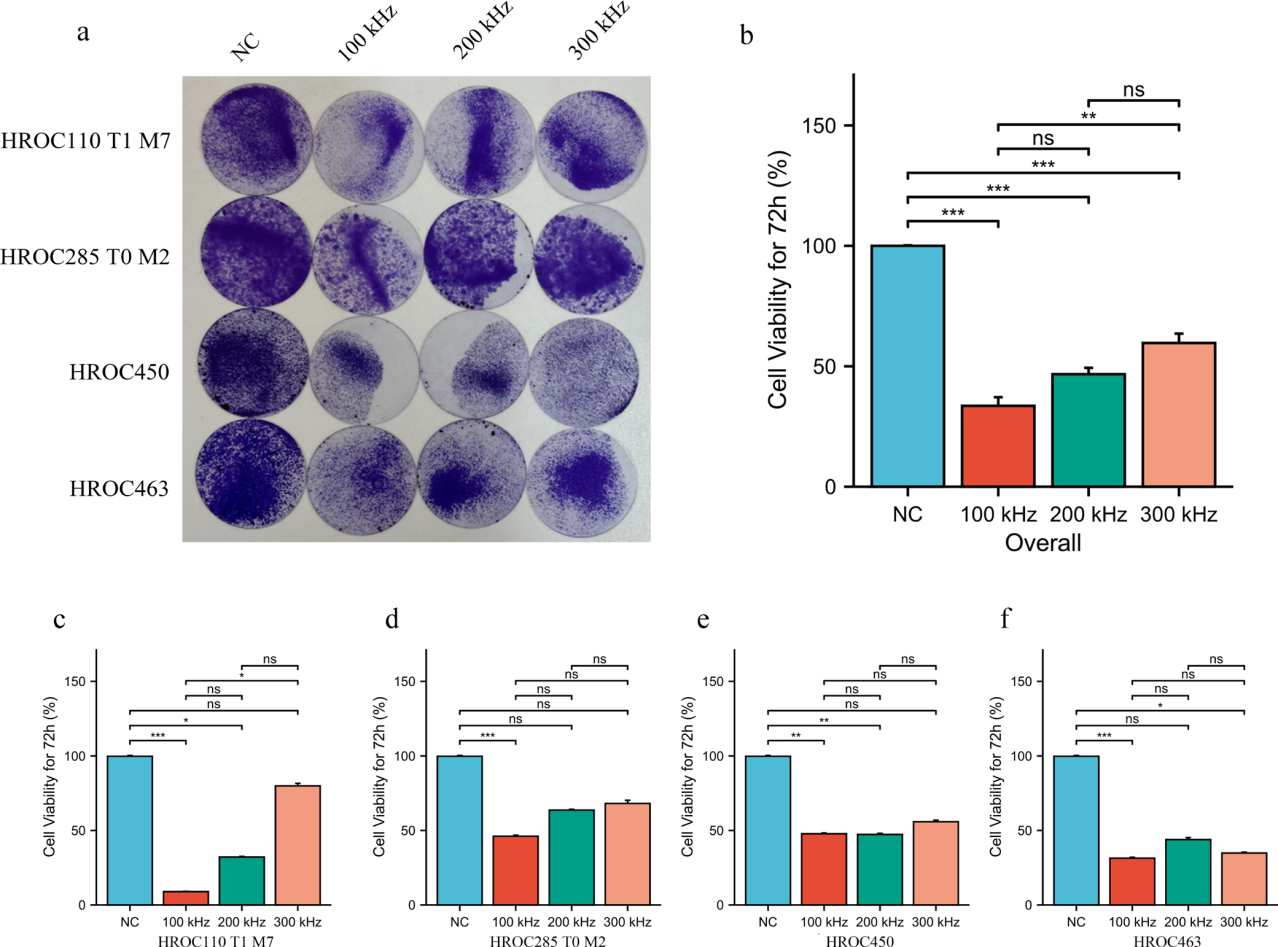
Effect of different frequencies on cell viability. (a, b) Effect of TTFields at 100 kHz, 200 kHz, and 300 kHz on cell viability in four CRC cell lines. TTFields were applied continuously for 72 h and at all three frequencies resulted in a reduction in cell viability. (c, d) Cell viability of MSI cell lines (HROC110 T1 M7 and HROC285 T0 M2) following TTFields treatment. (e, f) Effect of TTFields at various frequencies on MSS cell lines (HROC450 and HROC463). All data presented were obtained from at least three biological replicates. NC, Normal control; *p < 0.05, **p < 0.01, ***p < 0.001.

The overall analysis indicated that TTFields at all three frequencies, including 100 kHz, 200 kHz, and 300 kHz, effectively reduced cell viability. Of these frequencies, 100 kHz was observed to have a significantly superior effect compared to the other two frequencies (Figure 1a,b). Specifically, in MSI cell lines HROC110 T1 M7 and HROC285 T0 M2, the 100 kHz TTFields treatment exhibited the most pronounced suppression of cell viability, with a substantial reduction in cell survival compared to the control groups (*p* < 0.001). In contrast, while 200 kHz and 300 kHz TTFields also reduced cell viability, their effects were less pronounced than those of 100 kHz (Figure 1c,d). In the case of the two MSS cell lines HROC450 and HROC463, while TTFields treatment at different frequencies resulted in a significant decrease in cell survival compared to the control group, no significant differences were observed between the different frequencies (Figure 1e,f).

### 3.2 Effects of Treatment Duration on TTFields Efficacy

**FIGURE 2:**
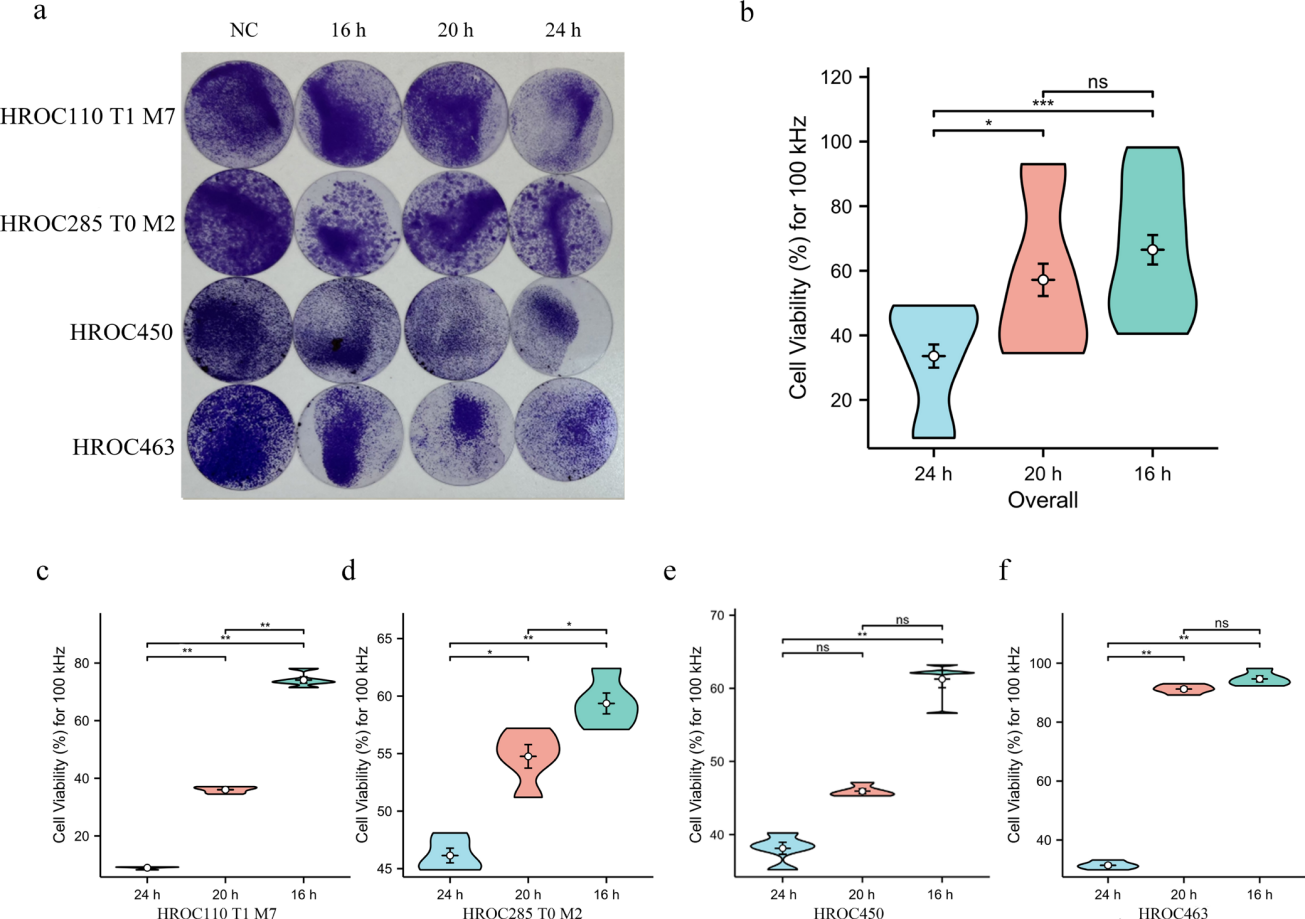
Effect of different exposure durations on cell viability. (a, b) Impact of TTFields at 100 kHz with varying daily exposure durations on cell viability in CRC cell lines. (c–f) Cell viability for the four cell lines HROC110 T1 M7 (c), HROC285 T0 M2 (d), HROC450 (e), and HROC463 (f). All data presented were obtained from at least three biological replicates. *p < 0.05, **p < 0.01, ***p < 0.001.

The impact of different treatment durations (16, 20, and 24‐h per day) on cell viability was assessed under a 100 kHz frequency electric field. In general, the daily treatment for 24‐h with TTFields resulted in the most substantial reduction in cell viability across all four CRC cell lines. No statistically significant differences in cell viability reduction were observed between the 16‐h and 20‐h treatment groups (Figure 2a,b). Specifically, the 24‐h/day TTFields treatment resulted in the greatest decrease in cell viability in HROC110 T1 M7 (Figure 2c; *p* < 0.01), HROC285 T0 M2 (Figure 2d; *p* < 0.01), HROC450 (Figure 2e; *p* < 0.01), and HROC463 (Figure 2f; *p* < 0.01). As the treatment duration was reduced to 20‐h/day and 16‐h/day, there was a significant increase in cell viability.

## 4 Discussion

Previous studies on TTFields have proven efficacy and safety in various cancers [9, 10, 11, 12, 13, 14, 15], leading to FDA approvals for glioblastoma multiforme and malignant pleural mesothelioma, with additional approvals pending. A significant factor influencing TTFields' effectiveness is the applied frequency, which varies by cancer type and affects tumor cell response. Mumblat et al. [16] reported that 150 kHz demonstrated the highest cytotoxicity in malignant pleural mesothelioma cells. However, Hoa et al. [17] found that for HeLa cells, 100 kHz exhibited greater cytotoxicity compared to 200 kHz. These findings highlight the necessity of optimizing TTFields frequency for different malignancies to maximize therapeutic benefit.

In this study, we investigated low‐passaged patient‐derived CRC cell lines to establish crucial baseline data for optimizing TTFields in CRC treatment. We evaluated the effects of three frequencies—100 kHz, 200 kHz, and 300 kHz—on the cell lines, revealing that 100 kHz was particularly effective in reducing cell viability in MSI CRC cell lines, while the impact of different frequencies on MSS cell lines was less pronounced. This suggests potential biological differences in TTFields sensitivity between MSI and MSS subtypes, underscoring the importance of frequency optimization to enhance therapeutic efficacy.

Another critical factor influencing the efficacy of TTFields is the duration of treatment. The present configuration of TTFields clinical devices necessitates continuous wear, and clinical evidence suggests enhanced effectiveness when used for more than 18 h per day [11]. However, this requirement can be challenging for patients, who must tolerate the device's use over extended periods. Consequently, a primary objective of TTFields research is to identify methods to preserve antitumor efficacy while concurrently reducing exposure duration. In our study, we assessed various treatment durations and found that extended treatment durations, particularly a 24‐h/day regimen, were associated with the most significant reduction in cell viability. This finding underscores the imperative for the development of a feasible and effective protocol for TTFields treatment duration and frequency, with the objective of enhancing patient compliance and optimizing treatment outcomes.

In conclusion, our study demonstrates that TTFields effectively reduce cell viability in CRC, thereby reinforcing their potential as a treatment option. However, further research is necessary to clarify the underlying mechanisms of TTFields action and explore the integration of TTFields with existing CRC therapies to assess potential synergistic effects, ultimately refining its clinical application and improving patient outcomes.

## Author Contributions

**Menglan Liu:** investigation, writing – original draft, data curation. **Zhaoran Su:** investigation, writing – original draft, writing – review and editing, validation, formal analysis. **Paul‐Philipp Hagemann:** investigation. **Malte Fischer:** investigation. **Michael Linnebacher:** conceptualization, writing – review and editing, formal analysis, supervision, resources.

## Ethics Statement

The authors have nothing to report.

## Conflicts of Interest

The authors declare no conflicts of interest.

## Data Availability

All original data and analysis results can be found within this study. For further information or detailed data, please contact Michael Linnebacher (michael.linnebacher@med.uni-rostock.de. Tel: +49381494146043).
